# Editorial: Mitochondrial control of cell fate

**DOI:** 10.3389/fcell.2023.1302075

**Published:** 2023-10-11

**Authors:** Guohua Gong, Huiliang Zhang, Stephen C. Kolwicz

**Affiliations:** ^1^ School of Laboratory Medicine and Life Sciences, Wenzhou Medical University, Wenzhou, China; ^2^ Shanghai East Hospital, Institute for Regenerative Medicine, Tongji University, Shanghai, China; ^3^ Department of Pharmacology and Toxicology, University of Arkansas for Medical Sciences, Little Rock, United States; ^4^ Heart and Muscle Metabolism Laboratory, Department of Health and Exercise Physiology, Ursinus College, Collegeville, PA, United States

**Keywords:** mitochondrial biogenesis, mitophagy, dynamics, cell fate, homeostasis

## Introduction

Mitochondria play a critical role in cells, not only through the regeneration of ATP, but via other cellular processes including calcium hemostasis, redox, and apoptosis (Li et al., 2022). Mitochondrial function is easily disturbed by environmental changes, such as nutrient restrictions, calcium overload, and oxidative stress (Schmidt et al., 2017; Mirza et al., 2021). Dysfunctional mitochondria have been implicated in the pathogenesis of various diseases, such as cancer, heart failure, and neurodegenerative diseases (Li et al., 2020; Ahn et al., 2021). Therefore, the maintenance of mitochondrial function and homeostasis are essential in the determination of cell fate (Markaki and Tavernarakis, 2020). Mitochondrial homeostasis is coordinately regulated by mitochondrial dynamics (i.e., fusion and fission), mitochondrial trafficking, mitochondrial autophagy (i.e., mitophagy), and mitochondrial biogenesis (Ma et al., 2020). However, the molecular mechanisms underlying mitochondria involvement in signaling and regulation of cell fate require further elucidation. Understanding the regulatory mechanisms of mitochondrial processes is also important in exploring mitochondrial-related diseases, in an effort to develop promising treatments. This Research Topic is comprised of 2 original research articles and 4 review articles, focused on how defects in regulation of mitochondrial signaling and function play a role in the determination of cell fate and disease.

## Mitochondrial dynamics

The results of the original study by Li et al. demonstrated that the function of the mitochondrial fusion protein, mitofusion 2 (MFN2), is determined by the phosphorylation state of three amino acids: T111, S378, and S442. The phosphorylation status at different sites variably affected MFN2 function. While non-phosphorylated MFN2 is responsible for mitochondrial fusion, mimicking phosphorylation on T111, S378 or S442 of MFN2 enhanced binding affinity, recruited parkin to mitochondria, and initiated mitophagy. Specifically, the mitochondrial fusion function of MFN2 was disabled by phosphorylation of MFN2 S378, while phosphorylation of S442 was necessary for mitochondrial Parkin translocation. Therefore, PINK1-mediated phosphorylation at T111, S378 or S442 is dependent and sufficient to switch MFN2 from fusion protein to mitophagy effector.

## Mitochondrial biogenesis

Two reviews included in this Research Topic delve into the machinery of mitochondrial biogenesis. Chen et al. highlight the role of PGC-1α, the master regulator of mitochondrial biogenesis, in the development of mitochondrial dysfunction and the contribution to heart failure. PGC-1α is involved in maintaining cardiac mitochondrial quality via regulation of mitochondrial biogenesis, mitochondrial dynamics, and mitophagy. PGC-1α activity can be enhanced by the phosphorylation of MAPK, AMPK, or the deacetylation of SIRT1. Since PGC-1α levels fluctuate in response to the development of heart failure, targeting PGC-1α activity appears as a potential therapeutic strategy.

In their review, Xu et al. discuss the role of transient receptor potential vanilloid 1 (TRPV1), located on the sarcoplasmic reticulum (SR), in the regulation of mitochondrial biogenesis in skeletal muscle. Activation of TRPV1 by high temperatures (40°C–45°C) leads to Ca^2+^ release from the SR to the cytoplasm. Mitochondrial biogenesis can be enhanced by long-term activation of TRPV1 through the Ca^2+^-CaMKII-p38 MAPK-PGC-1α signaling axis in skeletal muscle. Finally, persistent exposure of TRPV1 to excessive cellular Ca^2+^ stimulation can attenuate its activity.

Generally, mitochondrial biogenesis is considered a good actor, however, genetic PGC-1α overexpression does not offer a protective effect, causing abnormality of mitochondrial ultrastructure and impairment. Further exploration of the optimal dose and time course of PGC-1α, to achieve an ideal therapeutic effect, remains important.

## Mitophagy

Mitophagy plays a core role in mitochondrial health. The original work of Sun et al. identified hemocyte mitophagy in Pacific oyster *Crassostrea gigas* (*C. gigas*) after an immune challenge. Injection of the pathogen, *Vibrio splendidus,* induced the JC-1 monomerization, the cleavage of CgLC3, the colocalization of LC3 with mitochondria, and autophagy in oyster hemocytes of *C. gigas*. In addition, tissue analysis identified 10 mitophagy–related genes in *C. gigas* that have domains similar to homologs in mammals and yeast and include NIX, FUNDC1, PHB2, Cardiolipin, P62, VDAC2, MFN2, PARL, MPP, and OPTN, Overall, these results confirmed that the presence of the complete mitophagy pathway in mollusks. Furthermore, the regulation of mitochondrial function in *C. gigas*, particularly through mitophagy, may be important in the protection against pathogenic infection.

## Mitochondrial transfer

As a highly dynamic organelle, mitochondria can perform intercellular transfers to regulate cell homeostasis. Pang et al. reviewed the performance of macrophages in mitochondrial transfers. Healthy mitochondria transferred to recipient cells can promote the survival of cells by recovering mitochondrial function. Unhealthy mitochondria can be reutilized by trans-mitophagy degradation or extruded by extracellular vesicles, known as migrasomes, after transferring to recipient macrophages. Activated monocytes can deliver mitochondria-related damage-associated molecular patterns (DAMPs) including mitochondrial membrane components and 16 S mitochondrial ribosomal RNA to active macrophages. Therefore, mitochondrial transfers could function as immune signals. The regulation of mitochondrial transfers between macrophages and their surrounding cells in the control of cell fate is a promising therapy for various diseases, including cardiovascular diseases, inflammatory diseases, obesity, and cancer.

## Mitochondrial metabolism

Metabolism is a core process of mitochondria, which depends on mitochondrial homeostasis. The review from Persad et al. summarized the current knowledge regarding the effects of metabolic maturation of mitochondria on cardiomyocyte cell fate. The switch from primarily glycolytic metabolism to mitochondrial oxidative metabolism is essential in controlling cardiomyocyte maturation. The shift in metabolism is paralleled by changes in mitochondrial homeostasis and an increase in fusion and mitochondrial biogenesis. Mitochondrial biogenesis driven by PGC-1α can be activated by metabolic intermediates and other metabolites. The metabolic role of ketone bodies and glutamine as they apply to the physiological functioning of the heart will be particularly important for understanding the changes from fetal to newborn as well as for applications in regenerative medicine.

## Conclusion

**FIGURE 1:**
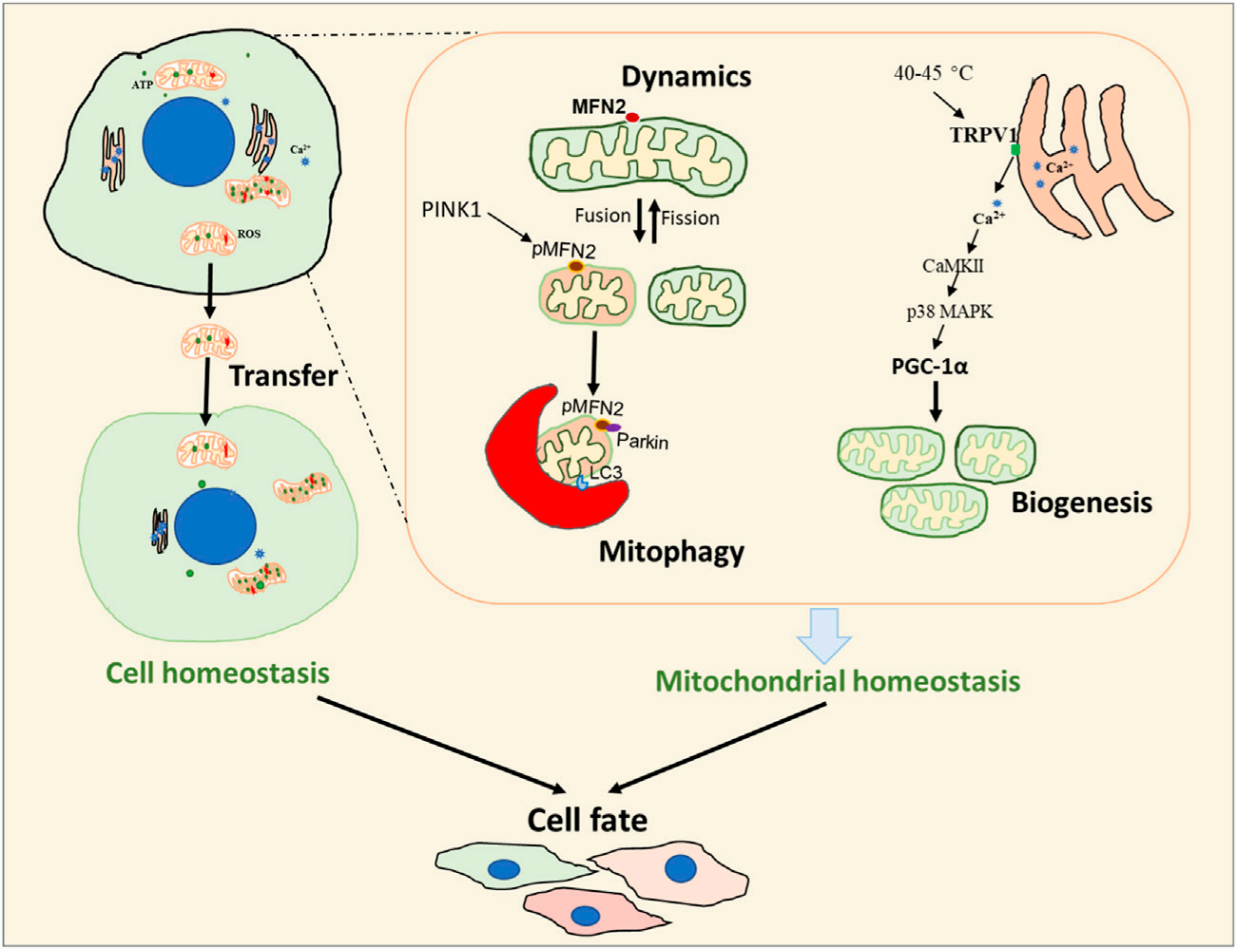
Overview of the metabolic processes involved in cell fate. Cell fate is regulated by both cellular and mitochondrial homeostasis. Mitochondrial transfer can be important in the determination of cellular homeostasis while the processes of mitochondrial dynamics, mitophagy, and biogenesis are critical in maintaining mitochondrial homeostasis.

In summary, changes in mitochondrial homeostasis and energy metabolism play a pivotal role in determining cell fate (Figure 1). This Research Topic highlights some of the current research that explores the role of mitochondrial health and the physiological and pathological processes involved in the control of cell fate. Further efforts focused on the elucidation of specific mitochondrial processes that determine cell fate may facilitate mitochondria targeting strategies to treat various diseases.
